# Artificial neural network and SARIMA based models for power load forecasting in Turkish electricity market

**DOI:** 10.1371/journal.pone.0175915

**Published:** 2017-04-20

**Authors:** Ömer Özgür Bozkurt, Göksel Biricik, Ziya Cihan Tayşi

**Affiliations:** Computer Engineering Department, Yıldız Technical University, İstanbul, Turkey; Chongqing University, CHINA

## Abstract

Load information plays an important role in deregulated electricity markets, since it is the primary factor to make critical decisions on production planning, day-to-day operations, unit commitment and economic dispatch. Being able to predict the load for a short term, which covers one hour to a few days, equips power generation facilities and traders with an advantage. With the deregulation of electricity markets, a variety of short term load forecasting models are developed. Deregulation in Turkish Electricity Market has started in 2001 and liberalization is still in progress with rules being effective in its predefined schedule. However, there is a very limited number of studies for Turkish Market. In this study, we introduce two different models for current Turkish Market using Seasonal Autoregressive Integrated Moving Average (SARIMA) and Artificial Neural Network (ANN) and present their comparative performances. Building models that cope with the dynamic nature of deregulated market and are able to run in real-time is the main contribution of this study. We also use our ANN based model to evaluate the effect of several factors, which are claimed to have effect on electrical load.

## Introduction

Deregulation in Turkish Electricity Market has started in 2001 and liberalization will be completed in a few years with the removal of the consumption limits of eligibility for consumers to choose their distributing company. This will cause distributors to offer prices as low as possible to attract new subscribers, while preserving the profitable level. Currently, distribution companies trade with manufacturers over the prices that are determined daily by EPIAS [1]. In the near future, as the market becomes fully deregulated and competitive, correct moves in the market will depend on the precision of the expected electricity cost.

Load is a fundamental and vital information for power generation facilities and traders, especially in production planning, day-to-day operations, unit commitment and economic dispatch. Load forecasting is done in different intervals according to requirements: long term load forecasting covers one to several years for plant and infrastructure investment decisions; mid-term load forecasting covers a few days to a few months for maintenance scheduling and negotiation of forward contracts; short term load forecasting (STLF) covers one hour to a few days for real time generation control, security analysis and energy transaction planning [2]. STLF can be nationwide [3], regional [4] or for microgrids [5]. Well known rules of supply-demand balance are also valid in electricity markets: price increases during the hours of higher demand and goes down during the hours of lower demand such as nights, weekends and holidays. Demand is shaped hourly and it is impossible to start or stop production instantaneously in a huge power plant; therefore, production planning is mostly done in daily basis. Thus, STLF plays a crucial role for managing operations in electricity markets.

With the deregulation of electricity markets, a variety of STLF models are developed. These models include multi linear regression [6], Box-–Jenkins method and other derived autoregressive models [7], artificial neural networks (ANNs) [8], fuzzy logic systems [9], Kalman Filter models [10] and hybrid models [11, 12]. Relationship between external factors and electrical load is not only quite complex but also nonlinear. This nature of load makes it difficult to predict future values with parametric modeling methods such as time series and linear regression analysis. Parametric methods require making assumptions on the rules of underlying system. On the other hand, ANNs require minimum number of assumptions to find out the relation between input and the output. For non-linear multivariate problems with large datasets, ANN is known to exhibit a much higher performance and therefore, seems to be appropriate for STLF. Contrariwise, autoregressive models sometimes outperform ANN based models due to seasonality effect.

Medium and long term forecasts are done using collected historical load data, weather data, the number of consumers in different classes, number and characteristics of appliances at the region, population data, electrical equipment sales and estimations of these for the interval to be forecasted [13]. On the other hand, short term forecasts use historical load, price and weather data. Introducing seasonality effect by using day of week, hour of the day and holiday information as input has shown to increase performance [14].

Deregulation of the Turkish Electricity Market is still in progress with new rules being effective. Earlier studies on Turkish Electricity Market are done in regional basis and span the period before the actual deregulation. Also there is a growing trend to use intermittent sources such as wind and solar energy to produce electricity due to the environmental concerns. However, irregular nature of these resources increases the degree of uncertainty of electrical load. Our primary motivation is to create an accurate STLF system for current Turkish Electricity Market, since both deregulation and the use of intermittent sources have changed the dynamics of the market.

The literature review on STLF shows that two main streamlines exist. The first group consists of regression [15–17] and time series methods [7, 18–23], where the performances are given in Mean Absolute Percentage Error (MAPE) and vary between 1.40% and 7.0%. The second group of studies are either ANN based [8, 24–28] or have some extensions and modifications to ANN, which are referred as hybrid solutions [6, 12, 29–36]. All these modifications tend to increase forecast performance, and this group of studies report MAPE values between 0.98% and 14.0%. We have to note that all these mentioned MAPE values are not standardized, changing from only one-hour-ahead forecasts to weekly mean values. Besides, the nature of the electricity markets used in these studies directly affects the forecast performances.

STLF studies for Turkish market are very limited. Filik *et al.* develop a statistical model to forecast short, medium and long term load for regulated Turkish Market. The success of the model for short term is given as 5.74% MAPE [37, 38]. Topalli *et al.* work on Turkish load data of year 2001 and develop an ANN model after clustering the data according to its characteristics. Their model outperform Autoregressive Moving Average (ARMA) model developed for benchmarking and achieve 1.51% weighted average MAPE [39, 40]. Yasin *et al.* compare performance of ANN and SVM based methods for STLF using calendar and temperature data of three major cities. They report MAPE values ranging from 2.0% to 3.55% determined by the season [41]. Cevik and Cunkas compare performance of ANN and Adaptive Neuro Fuzzy System (ANFIS) methods using load data of Turkish Market between 2009 and 2011. They report 1.85% to 2.02% MAPE values for ANN and ANFIS respectively [42].

As we reported above, the studies on Turkish Electricity Market are inadequate. First, they are outdated and do not fit to the deregulating structure of the Market. Second, they propose one method and lack on presenting comparisons. Our motivation is to fill this gap by proposing two separate models, one based on Seasonal Autoregressive Integrated Moving Average (SARIMA) and the other based on ANN. There are several factors including weather, currency, and price, which are believed to have effect on load. However, to the best of our knowledge there are limited number of studies investigating this subject and scope of these studies is limited to weather forecasts as in [43–46]. Another motivation for our work is investing the correlation between these factors and load by using real data spanning over two years.

Our contribution is three folded. First, while building our proposed systems, we used recent deregulated market data, which reflect the dynamic nature of Turkish Electric Market. Secondly, in most of the proposed STLF systems, successive one hour is predicted using previous actual values of inputs. This approach is not suitable to make a weekly prediction in real-time, since it requires actual values to be known beforehand. Our proposed systems are based on weekly predictions and able to forecast 168 hours ahead. Finally, on contrary to existing studies, we performed extensive test cases by using a week from each month of the year. Thus, we obtained fair and unbiased results, which includes effects of special days and seasons.

The rest of this paper is organized as follows. Details of the methods that we used to create our models, are given in Methods. Case studies and detailed discussion on experimental results are presented in Experimental results. Finally, we conclude the paper and provide guidelines for future work.

## Methods

Electrical load is a typical time series, since it consists of successive hourly measurements. Such time series data occur naturally in many application areas including process control, forecasting in economics, marketing, population studies, biomedical science. In order to understand the characteristics of a physical system that creates the time series, time series analysis methods that use systematic approaches are employed [47]. An important part of time series analysis is forecasting, which focus on prediction of future events based on the information extracted from the time series. There are different approaches used in time series analysis to forecast short and long term future. We can categorize these methods as parametric and non-parametric methods.

### Parametric methods

Parametric methods that are used for time series forecasting include mathematical models such as Autoregressive (AR), Moving Average (MA), ARMA, Autoregressive Integrated Moving Average (ARIMA) and SARIMA. These models are used frequently in electrical load and price forecasting [48, 49].

All these methods employ a four step approach to create a model. First, model is formulated as a hypothesis. Then, a specific model is formed by selected variables based on observations. In the third step, model parameters are estimated by least-squares or maximum likelihood. At the final step, performance of the model is tested with selected variables and parameters. If the performance of the model meets predefined criteria, then the forecasting model is accepted. Otherwise, new parameters for model are estimated. This procedure is repeated until a set of model parameters that satisfies our predefined criteria is found [50].

In AR models, a series of previous values *Z*_*t*−1_, *Z*_*t*−2_, …*Z*_*t*−*p*_ are used to forecast the value *Z*_*t*_. An AR model can simply be defined as in Eq (1).

$$\begin{array}{c} Z_{t}=C+\phi_{1}Z_{t-1}+\phi_{2}Z_{t-2}+...+\phi_{p}Z_{t-p}+\epsilon_{t} \end{array}$$ (1)

Where *C* is a constant, *ϕ*_1_, *ϕ*_2_, *ϕ*_3_, …, *ϕ*_*p*_ are coefficients, *ϵ*_*t*_ is forecast error and *p* is the number of autoregressive terms. The formula above can also be written as in Eq (2).

$$\begin{array}{c} Z_{t}=C+\sum_{i=1}^{p}\phi_{i}Z_{t-i}+\epsilon_{t} \end{array}$$ (2)

MA models use average of subsequences. As the process in a time series goes on, each new observation is added to the average and the oldest observation is dropped. Mathematical definition of MA models is given in Eq (3).

$$\begin{array}{c} Z_{t}=\epsilon_{t}-\sum_{j=1}^{q}\theta_{j}\epsilon_{t-j} \end{array}$$ (3)

Where *θ*_*j*_ are model parameters and *ϵ*_*t*_ is error. *q* represents the number of moving average terms. ARMA models are formed by combining AR and MA models. An ARMA(*p*, *q*) model can be expressed as:

$$\begin{array}{c} Z_{t}=C+\sum_{i=1}^{p}\phi_{i}Z_{t-i}+\epsilon_{t}-\sum_{j=1}^{q}\theta_{j}\epsilon_{t-j} \end{array}$$ (4)

In practice, most of the time series are non-stationary. In order to fit a stationary model, it is necessary to remove non-stationary sources of variation. This can be done by differencing. Integrating ARMA(*p*, *q*) process to the *d*^*th*^ order creates a model that is capable of describing certain types of non-stationary series [51]. This model is called ARIMA and can be shown as ARIMA(*p*, *d*, *q*), where *d* is the number of nonseasonal differences needed for stationarity.

Time series may possess seasonal patterns such as daily, weekly, monthly, etc. In order to model such time series, SARIMA models can be used. A SARIMA model is an extended version of ARIMA model with additional seasonal terms and can be shown as ARIMA(*p*, *d*, *q*) × (*P*, *D*, *Q*)_*s*_, where *P* is the degree of seasonal AR model, *Q* is the degree of seasonal MA model, *D* is degree of seasonal integration, and *s* is the span of repeating seasonal pattern. Detailed discussion about variable selection of our SARIMA based model is given in Experimental results.

### Non-parametric methods

The methods discussed in the previous subsection rely on tuning the parameters of the defined model. On the other hand, due to the nature of the time series data, the coefficients and the constant can belong to an unknown distribution and may not be described with parameters. This situation especially arise from the non-stationary nature of the data. To overcome this problem, non-parametric forecasting methods are introduced [52–54].

**Fig 1:**
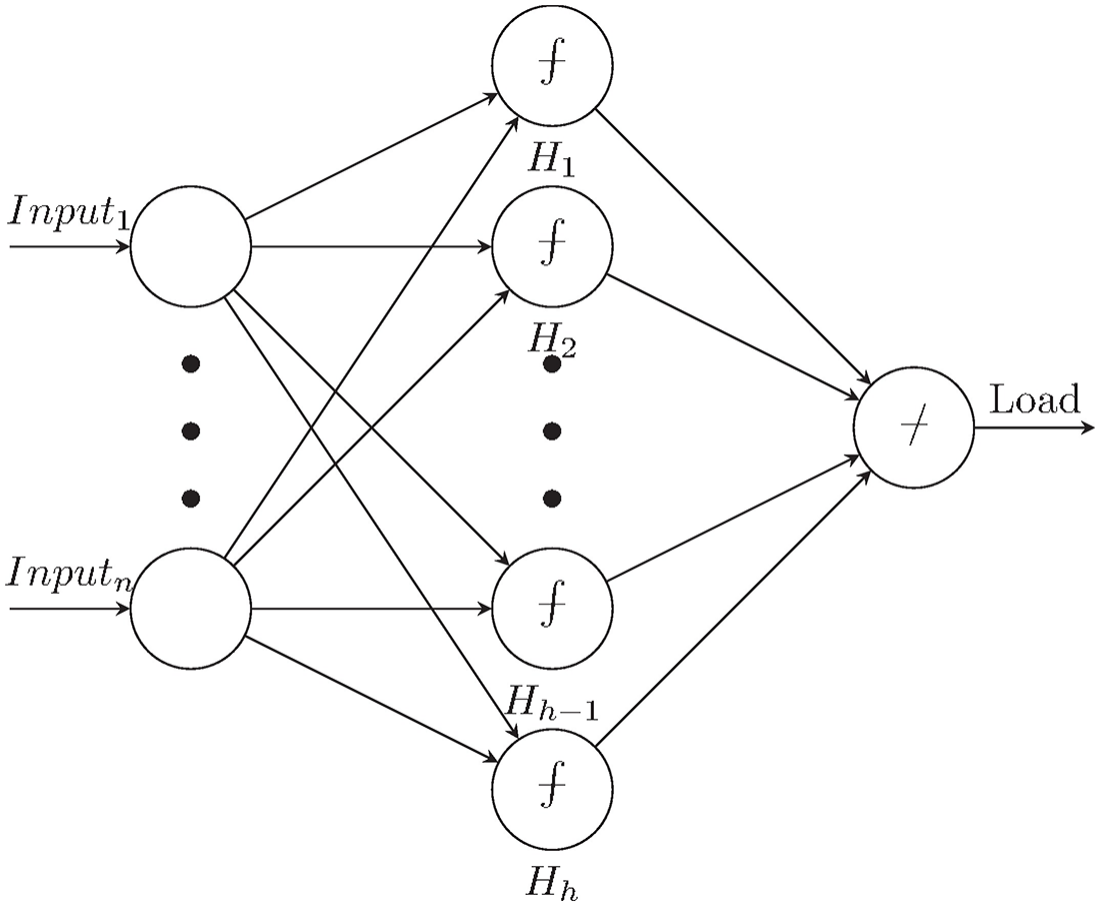
Feed-forward neural network design.

In early studies, non-parametric kernel estimators are used to adjust the coefficients of AR, MA, ARMA and ARIMA methods [55]. Later on, ANNs are used as non-parametric estimators for time series forecasting. ANN can fit a non-parametric and non-linear function, without guidance to time series data [56–58]. ANN is a biologically inspired machine learning method, which simulates the workflow of human neural system. However, the function approximation and learning algorithms differ from the way that the biological nerves behave. The underlying mechanism of ANNs is defining a function by means of weighted sum of several sigmoids. These sigmoid transfer functions are in fact the combining functions of all relevant explanatory variables. The weights of the sigmoids are determined with regard to the impacts of the input variables and their interrelations, typically with gradient search algorithms. This structure enables the network to fit a non-linear function to the given data. This is achieved by the multi-layered topology, where the input layer normalizes and weighs the inputs, the hidden layer fits nonlinear function to the presented data through the transfer functions, and the output layer sums up the results. These layers consist of the processing units called neurons. The topology of ANN is formed by the weighted connection structure between the neurons. Multi-layered topology with direct weighted connections is known as the Feed Forward (FF) network and visualized in Fig 1. Besides FF, there are many ANN topologies presented in the literature, each having a different specific target according to the nature of the data and the problem. The most commonly used network topologies in short-term electrical load and price forecasting are discussed in [59]. In this study we used FF ANN as it is already shown that they perform better on forecasting [60].

In order to train a network for adapting to the introduced data for the desired output, the objective function must be minimized, using learning algorithms. Back Propagation (BP) is the most commonly used error distribution model in learning phase of ANNs. In order to minimize the objective function, or cost, different algorithms are presented. Starting from the slow converging gradient descent, each learning algorithm works fine on certain types of datasets or objectives. Scaled Conjugate Gradient (SCG), Levenberg-Marquardt (LM), Quasi-Newton (QN) and Bayesian Regulation (BR) are the examples of common learning algorithms used in ANNs. The LM algorithm works 10 to 100 times faster than BP [61]. This makes LM the most convenient learning algorithm in many tasks. Newton’s minimization function for vector *x* is

$$\begin{array}{c} \Delta x=-{\left[J^{\text{T}}\left(x\right)J\left(x\right)\right]}^{-1}J^{\text{T}}\left(x\right)e\left(x\right) \end{array}$$ (5)

where *J*(*x*) is the Jacobian matrix and *e*(*x*) is the error vector. The LM algorithm is in fact an update to Newton’s minimization, defined as:

$$\begin{array}{c} \Delta x=-{\left[J^{\text{T}}\left(x\right)J\left(x\right)+\mu I\right]}^{-1}J^{\text{T}}\left(x\right)e\left(x\right) \end{array}$$ (6)

and practically solves the situations where $J^{\text{T}}\left(x\right)J\left(x\right)$ can not be inverted. The *μ* coefficient adjusts the convergence speed of LM. While small values provide fast convergence, large values speeds down and turns the algorithm into the steepest descent. *μ* can be modified during learning phase, between iterations, to guarantee convergence.

Due to the nature of ANN, every feature vector can be presented as an input neuron. This structure enables us to form any subset of features that effect electrical load and use these as the inputs of the forecasting NN. The subsets of features and their impact on load forecast performance is discussed in Dataset section.

## Experimental results

We evaluated our power load forecasting models on data from deregulated Turkish Market. In this section, we present our dataset, performance evaluation metric and experimental setups of the selected methods.

### Dataset

Electrical load depends on several factors including calendar effect, consumption, electricity price, weather and currency. The effects of these factors can be explained as follows: Calendar effect shapes demand through working hours, holidays, and national or religious days. Consumption corresponds to the electricity demand of both industrial and residential consumers. Electricity price is shaped by both production and trading, and influences load. Weather conditions can change power demand. It is known that temperature, relative humidity, wind speed and direction are the most affectional weather parameters, since the usage of air conditioners or electrical heaters are directly related to these factors. Currency is another major factor because it directly affects the electricity production costs and cross-border electricity trade agreements.

Selecting the correct combination of input parameters is the key to create an effective electrical load forecasting system. In order to attain a good combination, data is collected from several sources related to the factors mentioned above. We evaluated the effects of these factors on load forecast performance using ANN and compared the results with SARIMA. Load, electricity price, and weather data are collected in hourly period between 01.01.2013 and 31.12.2014. Currency data is collected in daily basis. Using these data, we constructed our training and test sets. We established our test sets by selecting the last full week, starting from Monday, of the corresponding month in 2014. The data in the preceding 1, 3, 6 and 12 months of the test weeks are used for training. The training and test sets for the selected weeks are given in Table 1, with start and end dates.

**Table 1:** Start and end dates of the training and test periods for the selected test weeks.

Train set						Test set	
Start date							
Weeks	1 month	3 months	6 months	12 months	End date	Start date	End date
W1	20.12.2013	20.10.2013	20.07.2013	20.01.2013	19.01.2014	20.01.2014	26.01.2014
W2	17.01.2014	17.11.2013	17.08.2013	17.02.2013	16.02.2014	17.02.2014	23.02.2014
W3	24.02.2014	24.12.2013	24.09.2013	24.03.2013	23.03.2014	24.03.2014	30.03.2014
W4	21.03.2014	21.01.2014	21.10.2013	21.04.2013	20.04.2014	21.04.2014	27.04.2014
W5	19.04.2014	19.02.2014	19.11.2013	19.05.2013	18.05.2014	19.05.2014	25.05.2014
W6	23.05.2014	23.03.2014	23.12.2013	23.06.2013	22.06.2014	23.06.2014	29.06.2014
W7	21.06.2014	21.04.2014	21.01.2014	21.07.2013	20.07.2014	21.07.2014	27.07.2014
W8	25.07.2014	25.05.2014	25.02.2014	25.08.2014	24.08.2014	25.08.2014	31.08.2014
W9	22.08.2014	22.06.2014	22.03.2014	22.09.2014	21.09.2014	22.09.2014	28.09.2014
W10	20.09.2014	20.07.2014	20.04.2014	20.10.2014	19.10.2014	20.10.2014	26.10.2014
W11	25.10.2014	24.08.2014	24.05.2014	24.11.2014	23.11.2014	24.11.2014	30.11.2014
W12	22.11.2014	22.09.2014	22.06.2014	22.12.2014	21.12.2014	22.12.2014	28.12.2014

The hourly load data and market clearing prices for Turkish Market are gathered from EPIAS [62]. Using this data, we calculated hourly lagged load data that includes the previous hour load, the load at the same hour on previous day, on previous week, and average load on last 24 hours. Besides load data, we prepared calendar data by marking weekdays, weekends, Turkish national and religious holidays.

We collected weather data for the major cities of Turkey from Turkish State Meteorological Service [63]. After analyzing their impact on load forecast performance, we selected four prominent cities: İstanbul, Ankara, İzmir and Antalya. The weather data consist of hourly temperature and humidity values for these city centers.

Most of the wholesale trade in Turkish Electricity Market is made using foreign currencies. Thus, foreign exchange currency rates for Euro and US Dollar are collected from the Central Bank of the Republic of Turkey archives [64]. Unfortunately, historical hourly rates for these currencies are not provided in the records. Thus, we used daily currency exchange rates for every hour in a day.

**Fig 2:**
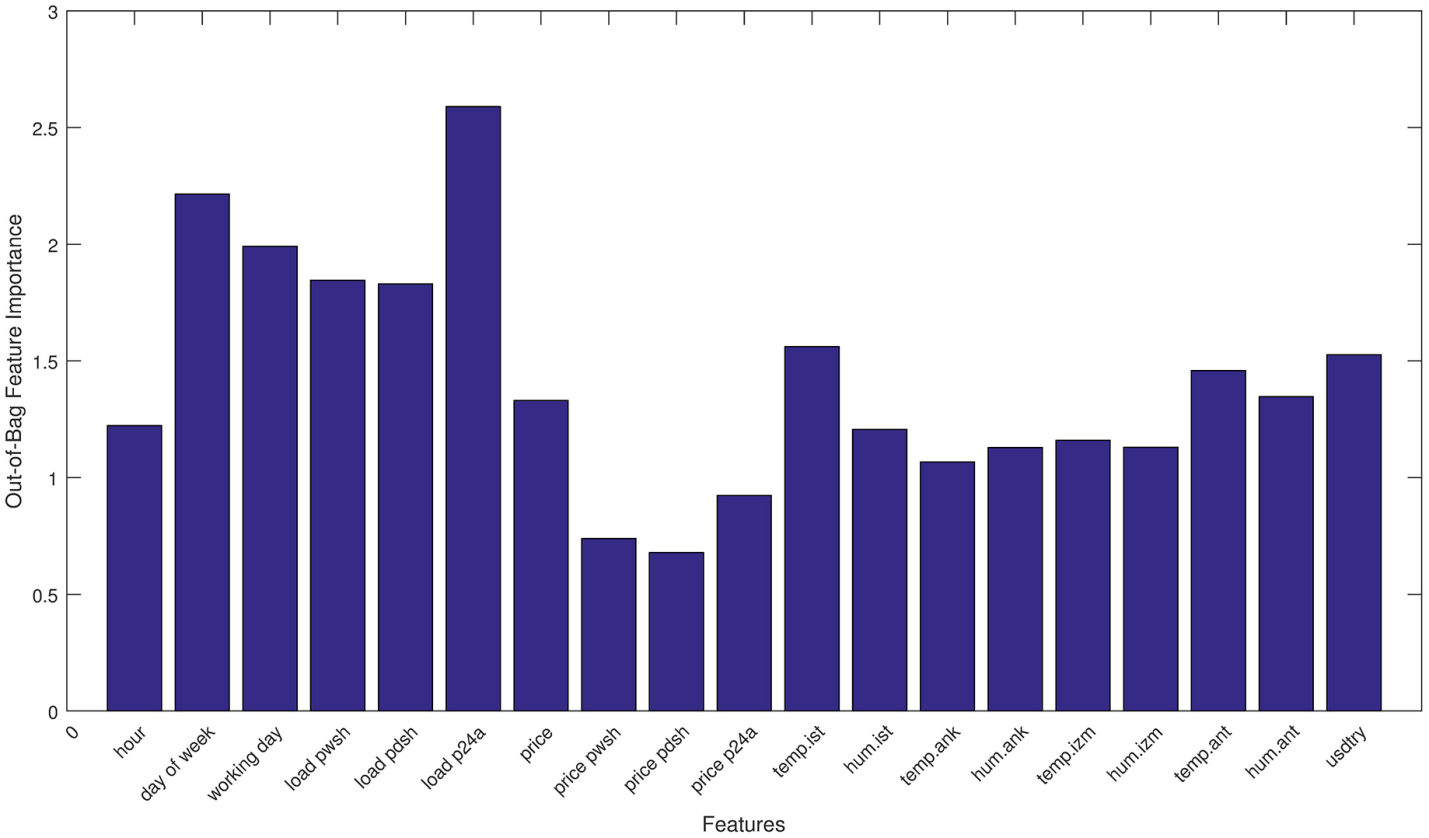
Comparison of feature importances on load.

A detailed description of our feature sets and the features within these groups are presented in Table 2. In order to evaluate the effect of these features on load, we analyzed their correlation coefficients with respect to the hourly load data. Based on the *p*-values matrix, we can easily say that there is a significant correlation between the selected input features and load. Besides statistical parameter observations, we estimate the importance of inputs using a bootstrap aggregated random-forest ensemble. Out-of-bag importance of the selected features are given in Fig 2, and it clearly shows that all these features have impact on load.

**Table 2:** Details of feature sets that are used in ANN based STLF model.

Feature Set	Included Features
Calendar Data (D)	Day of week
	Working day
	Holidays
Previous Load Estimation Plan (L)	Previous day same hour load
	Previous week same hour load
	Previous 24 hour average load
Electricity Price (P)	Previous market clearing price
	Previous day same hour price
	Previous week same hour price
	Previous 24 hour average price
Weather (W)	İstanbul temperature
	İstanbul relative humidity
	Ankara temperature
	Ankara relative humidity
	İzmir temperature
	İzmir relative humidity
	Antalya temperature
	Antalya relative humidity
Currency (C)	USD/TRY exchange rate

### Performance metric

In this study, we use Absolute Percentage Error (APE) and MAPE to measure the performances of the proposed approaches. APE is calculated by Eq (7) and is used to show the maximum and minimum forecast errors. On the other hand, MAPE gives an overall performance evaluation of proposed approaches. MAPE formula is given in Eq (8). In Eq (7), *LP*_*i*_ is the Load Estimation Plan value, that is the original value provided by EPIAS, whereas *LE*_*i*_ is the estimated Load at hour *i*. In Eq (8), *N* corresponds to total number of estimated hours.

$$\begin{array}{c} APE_{i}=\frac{|LP_{i}-LE_{i}|}{LP_{i}} \end{array}$$ (7)

$$\begin{array}{c} MAPE=\frac{1}{N}\sum_{i=1}^{N}APE_{i} \end{array}$$ (8)

### Creating SARIMA model

**Fig 3:**
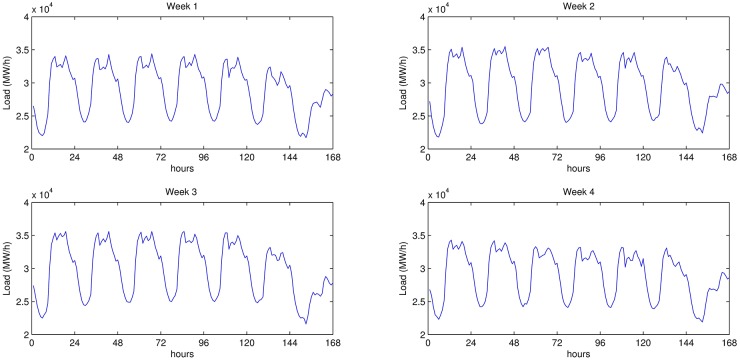
Power load in four consecutive weeks of March 2014.

Electrical load of four consecutive weeks on March 2014 is given in Fig 3. A close inspection of the figure shows a distinct weekly seasonal pattern, which electrical load possesses. Thus, in this study, we prefer to build a SARIMA model, which can be shown as ARIMA(*p*, *d*, *q*) × (*P*, *D*, *Q*)_*S*_. Determining the values of *p*, *q*, *d*, *P*, *Q*, and *D* plays a crucial role for creating a highly accurate SARIMA model. We used Econometrics Toolbox of Matlab to determine these values, and to estimate parameters of our SARIMA models.

**Fig 4:**
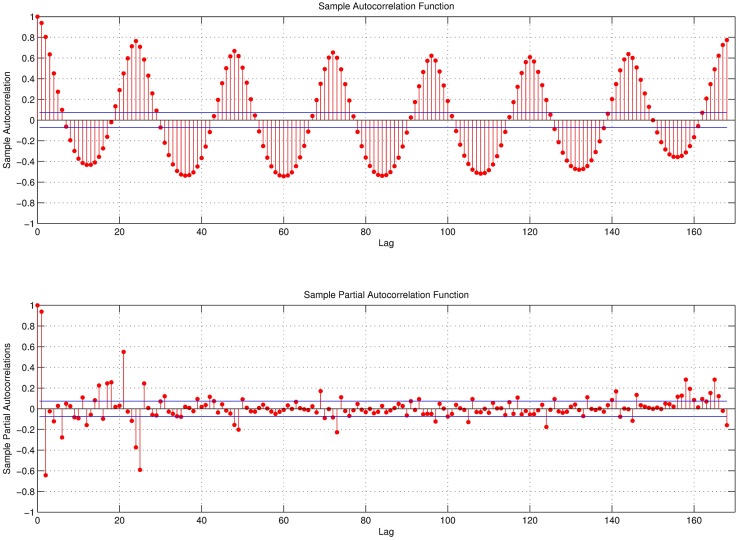
Autocorrelation function and partial autocorrelation function of load.

In most of the previous studies, variables of proposed ARIMA models are determined intuitively as in [18, 40, 65]. It is also possible to use Sample Autocorrelation Function (ACF) and Partial Autocorrelation Function (PACF) for determining *p* and *q* variables [7, 32]. ACF and PACF of the electrical load for March 2014 are given in Fig 4, which shows that there is a high correlation between the first few lags and the actual load. This figure also shows that *t* − 168^*th*^ lag has a high influence on the *t*^*th*^ hour. This also validates our assumption about the weekly seasonal characteristic of the electrical load.

In order to select degrees of *p* and *q* parameters, we employed Bayesian Information Criterion (BIC). We estimated several models with different *p* and *q* values. Then, for each estimated model, the log-likelihood objective function value is calculated. This value is then used to calculate the BIC measure of fit. In our study, we scanned a wide range of *p* and *q* values, and observed that the best fitted model is constructed when both *p* and *q* values are set to 1. BIC values of models with different *p* and *q* values are given in Table 3.

**Table 3:** BIC values of SARIMA models with different *p* & *q* values.

p	q											
	1	2	3	4	5	6	7	8	9	10	11	12
1	10632	10651	10651	10660	10656	10655	10664	10668	10684	10684	10717	10696
2	10646	11199	11211	11224	11207	11225	11202	11227	11277	11253	11254	11258
3	11085	11335	11495	11522	11497	11472	11504	11508	11538	11597	11596	11597
4	11097	11228	11575	11724	11685	11682	11696	11708	11744	11761	11814	11765
5	11273	11481	11574	11842	11829	11798	11809	11841	11877	11899	11900	11903
6	11290	11575	11527	11814	11960	11830	11911	11925	11952	12030	12048	11991
7	11338	11565	11621	11802	11980	12001	11922	11941	11992	12033	12039	12043
8	11363	11517	11673	11747	11975	12037	12006	11947	11999	12053	12066	12071
9	11347	11577	11686	11771	11949	12060	12054	12010	11937	12027	12080	12097
10	11387	11605	11674	11787	11899	12013	12051	12057	12021	11966	12043	12066
11	11351	11577	11634	11791	11852	11974	12032	12064	12059	11989	11949	12010
12	11388	11549	11606	11819	11850	11933	12022	12074	12076	12032	11988	11925

We also created another SARIMA model and selected its parameters intuitively. Our selection basically depends on the idea that electrical load on time *t* (*L*_*t*_) depends on the load on last three hours (*L*_*t*−1_, *L*_*t*−2_, *L*_*t*−3_), the load at same hour in previous day (*L*_*t*−24_), the load 48 (*L*_*t*−48_) hours ago and the load 72 (*L*_*t*−72_) hours ago. Variables used in these models are given in Table 4.

**Table 4:** Variables of both SARIMA models.

Model	Non-seasonal			Seasonal		
	AR Lags	d	MA Lags	AR Lags	D	MA Lags
BIC based model	1	0	1	1	0	1
Intuitive model	1, 2, 3, 24, 48, 72	0	1, 2	1	0	1

**Fig 5:**
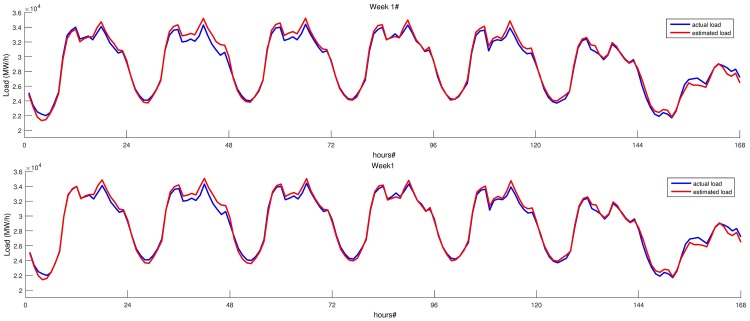
Load estimation of both SARIMA models for the last week of January 2014. Estimations for week 1, BIC based SARIMA model is shown in the upper part. Estimations of intuitive SARIMA model are given in the lower part.

**Fig 6:**
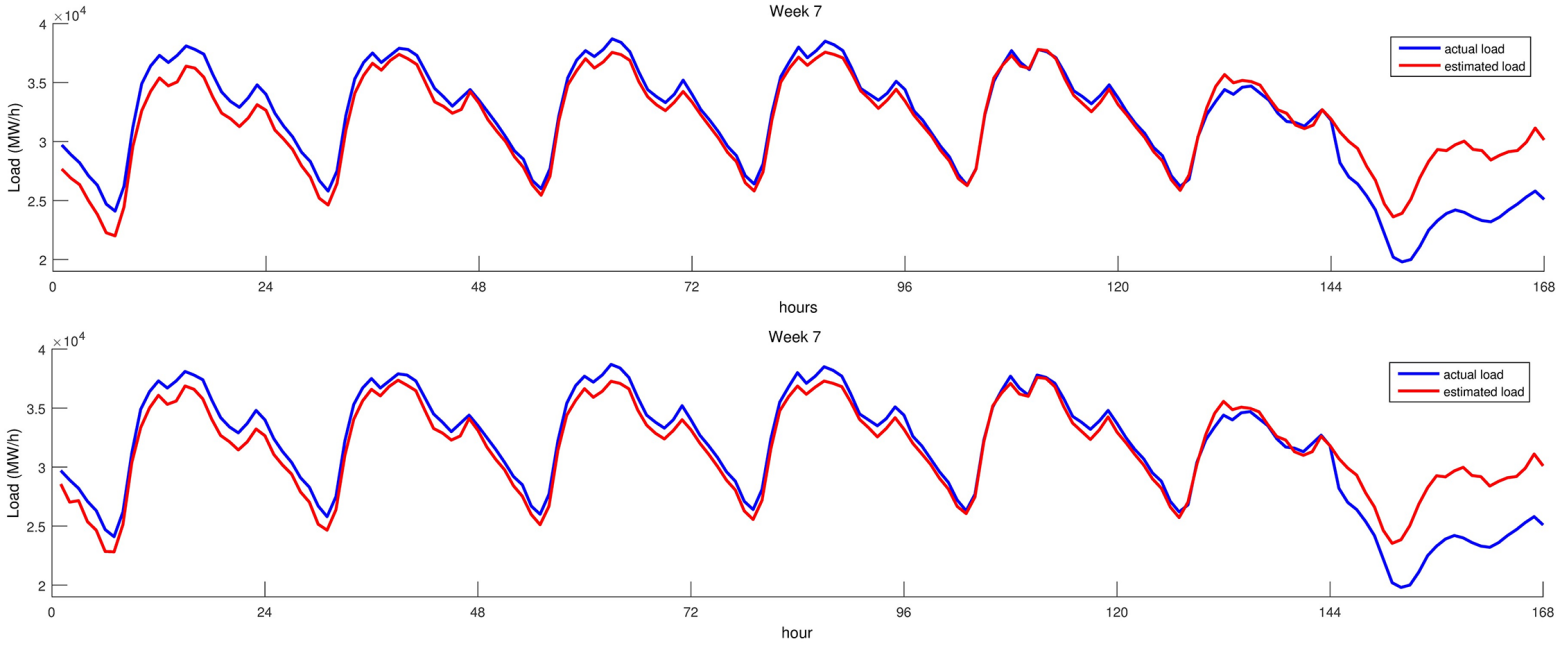
Load estimation of both SARIMA models for the last week of July 2014. Estimations for week 7, BIC based SARIMA model is shown in the upper part. Estimations of intuitive SARIMA model are given in the lower part.

We evaluated the performance of the proposed SARIMA models using one week of each month in 2014. Table 5 shows the MAPE values of both models for each week. Load estimations of both models and the actual load for the first test week is given in Fig 5. Figure clearly shows that both models perform well in weekdays. However, their accuracy is very low on weekends. This is due to fact that there is no information about weekdays or weekends supplied to both models. On the other hand, both models have relatively higher MAPE values on test week 7, that corresponds to July. Load estimated by our models and actual load for these weeks are given in Fig 6. Test week 7, which starts from July 21 and ends at July 27, overlaps with the Ramadan Feast Eve. Thus, last day of this week has higher error rate that increases overall MAPE value of the week.

**Table 5:** Comparison of proposed SARIMA models.

Weeks	SARIMA MODEL	
	Intuitive	BIC
W1	0.0124	0.0136
W2	0.0384	0.0238
W3	0.0212	0.0184
W4	0.0216	0.0257
W5	0.0253	0.0234
W6	0.0409	0.0374
W7	0.0501	0.0459
W8	0.0137	0.0195
W9	0.0325	0.0292
W10	0.0304	0.0271
W11	0.0296	0.0310
W12	0.0164	0.0175
Average	0.0277	0.0260

Overall performance of both models are very close. However, our first model, which was built using BIC, outperforms the intuitive model. Thus, we preferred to use BIC-based SARIMA model and hereafter SARIMA refers to our BIC-based model.

### Creating neural network model

The architectural properties of ANN directly effect the performance. In a FF network, most crucial properties are the hidden layer size, learning method and the length of training data. In order to successfully select and adjust these properties, we ran comparative tests on our dataset.

Choosing the number of hidden neurons is an important problem since there is not a certain way to determine it. Previous studies report that most widely used hidden neuron numbers are 2*n* + 1, (*n* + 1)/2, where *n* is the number of input neurons [66]. We observed that 2*n* + 1 hidden neurons worked best especially in shorter training datasets. It is also possible to use the trial and error approach to determine number of hidden neurons. Our trials showed that 20 hidden neurons produced better results in larger training datasets. A detailed comparison of hidden layer size effect on load forecast performance using LM is given in Table 6. Since highest forecast performance is expected using larger training data, we set our hidden layer to 20 neurons.

**Table 6:** Impact of hidden layer size on ANN performance, measured with MAPE (%). Smaller MAPE means higher forecast accuracy. The network is trained using LM. D refers to calendar data, L is previous load estimation plan, P is electricity price, W is weather and C is currency feature sets.

# of hidden neurons	Training dataset size	Feature set				
		DL	DLP	DLW	DLC	DLPWC
		(n = 6)	(n = 7)	(n = 14)	(n = 8)	(n = 17)
$\frac{n+1}{2}$	1 month	0.0282	0.0273	0.0364	0.0326	0.0437
	3 months	0.0267	0.0254	0.0278	0.0294	0.0324
	6 months	0.0252	0.0234	0.0269	0.0258	0.0268
	12 months	0.0194	0.0208	0.0218	0.0250	0.0229
2*n* + 1	1 month	0.0281	0.0330	0.0412	0.0538	0.0511
	3 months	0.0236	0.0262	0.0343	0.0287	0.0359
	6 months	0.0235	0.0347	0.0301	0.0239	0.0331
	12 months	0.0185	0.0184	0.0217	0.0200	0.0842
20	1 month	0.0318	0.0310	0.0404	0.0509	0.0429
	3 months	0.0323	0.0274	0.0330	0.0293	0.0380
	6 months	0.0218	0.0226	0.0303	0.0261	0.0304
	12 months	0.0180	0.0184	0.0217	0.0202	0.0227

**Fig 7:**
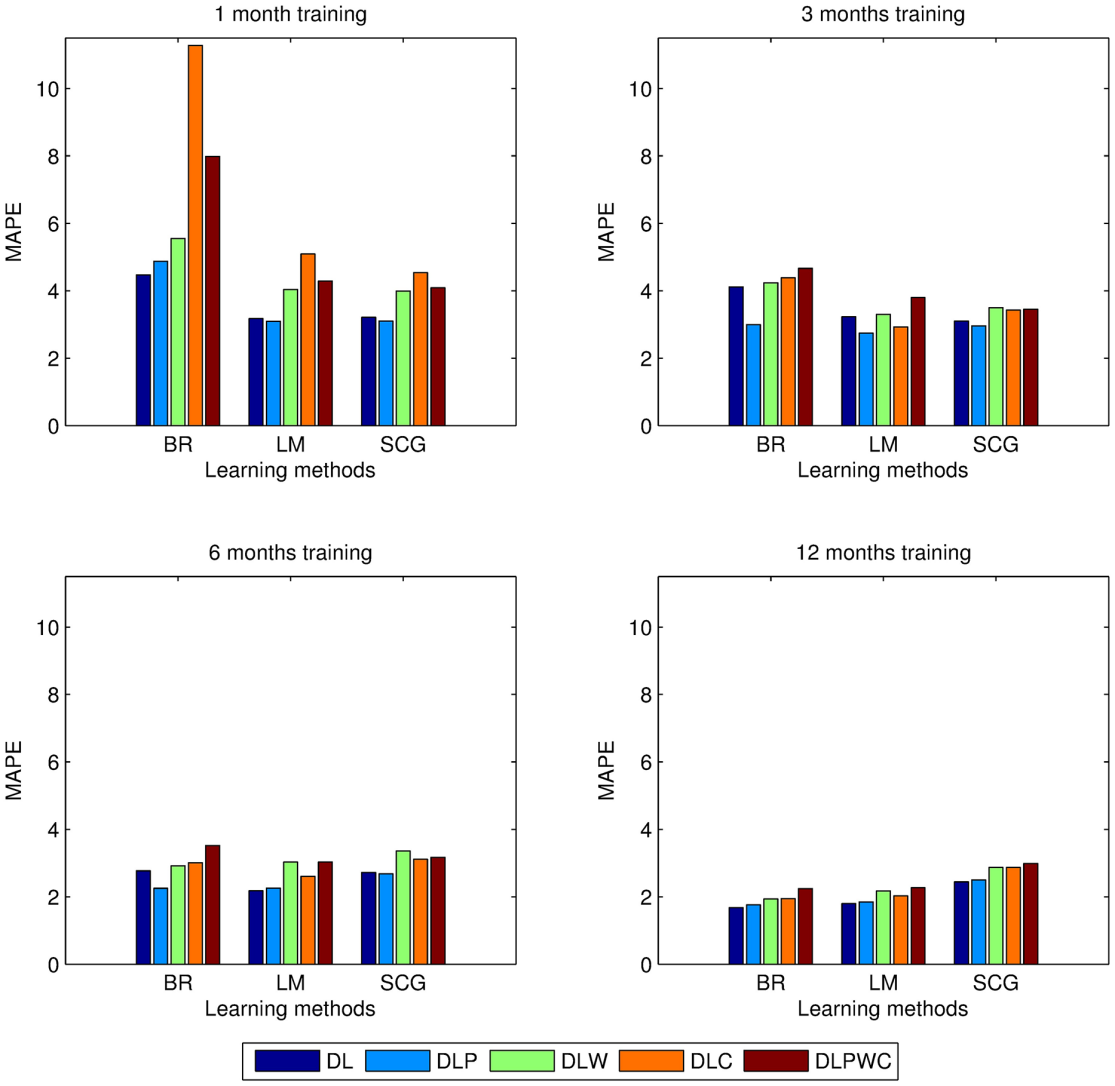
Performance comparison of NN learning methods across feature sets, measured with MAPE (%). Smaller MAPE means higher forecast accuracy. D refers to calendar data, L is previous load estimation plan, P is electricity price, W is weather and C is currency feature sets.

We discussed popular learning methods for training ANN in Methods. We compared three different learning methods, LM, BR and SCG using 20 hidden neurons. The impact of these learning methods across the feature sets, grouped by training dataset size, are compared in Table 7 and visualized in Fig 7. The comparison results showed that LM is the most convenient learning method that reasonably works good for every training dataset size. In addition, overall error decreases as the training dataset gets larger, regardless of learning algorithms.

**Table 7:** Learning method performance evaluation across different feature sets, grouped by training set length. Performance is measured with MAPE (%). Smaller MAPE means higher forecast accuracy. D refers to calendar data, L is previous load estimation plan, P is electricity price, W is weather and C is currency feature sets.

Training dataset size	Learning method	Feature set				
		DL	DLP	DLW	DLC	DLPWC
1 month	BR	0.0447	0.0487	0.0555	0.1128	0.0799
	LM	0.0318	0.0310	0.0404	0.0509	0.0429
	SCG	0.0321	0.0310	0.0399	0.0454	0.0409
3 months	BR	0.0412	0.0300	0.0424	0.0439	0.0467
	LM	0.0323	0.0274	0.0330	0.0293	0.0380
	SCG	0.0310	0.0296	0.0350	0.0343	0.035
6 months	BR	0.0277	0.0225	0.0292	0.0301	0.0352
	LM	0.0218	0.0226	0.0303	0.0261	0.0304
	SCG	0.0272	0.0268	0.0336	0.0311	0.0317
12 months	BR	0.0168	0.0176	0.0193	0.0195	0.0224
	LM	0.0180	0.0184	0.0217	0.0202	0.0227
	SCG	0.0244	0.0250	0.0287	0.0287	0.0298

Here we define the detailed parameters of our FF network, based on the determined hidden layer size and learning method above. Our input neurons vary from 6 to 19, depending on the feature set combinations of calendar data (D), previous load estimation plan (L), electricity price (P), weather data (W), and currency (C). The DL combination has 6 input neurons. DLP, DLW and DLC combinations have 10, 14 and 7 input neurons respectively. When all of the aforementioned features are used, the system has 19 inputs. We have a fully connected hidden layer with 20 neurons. The hidden neurons are activated with tansig transfer function. Our output neuron has linear activation function. Bias is introduced to both hidden and output neurons. Our model does not have input delays or layer delays. We measure the error on training with mean squared error, only with error minimization. LM training algorithm is used with 1000 maximum training epochs, 6 validation checks and *μ* beginning from 0.001, with 0.1 decrement and 10 increment ratios with 10,000 maximum limit. Usually, *μ* values converge to 10. We used Matlab Neural Network Toolbox in order to build and train the network.

Another focus point on the forecast performance of ANN is the impact of the feature sets. We evaluated the effect of these features by creating combinations of D, L, P, W, and C. The comparative results are given in Table 7. The *t*-tests proved that using DL, DP, DLW and DLPWC for load forecasting is statistically significant. On contrary to our expectations, currency has no positive effect on performance. This situation is clearly proved in our tests. Similarly, weather and price has minor positive effects. Calendar data and previous load values work well for load forecasting with adequate precision. We observed that an ANN with 20 hidden neurons, trained with DL of previous 12 months using LM learning algorithm, produced lowest MAPE. Our test results showed that using larger training dataset and simpler feature sets work better on load forecasting with ANN on Turkish Market.

### Comparative discussion

**Fig 8:**
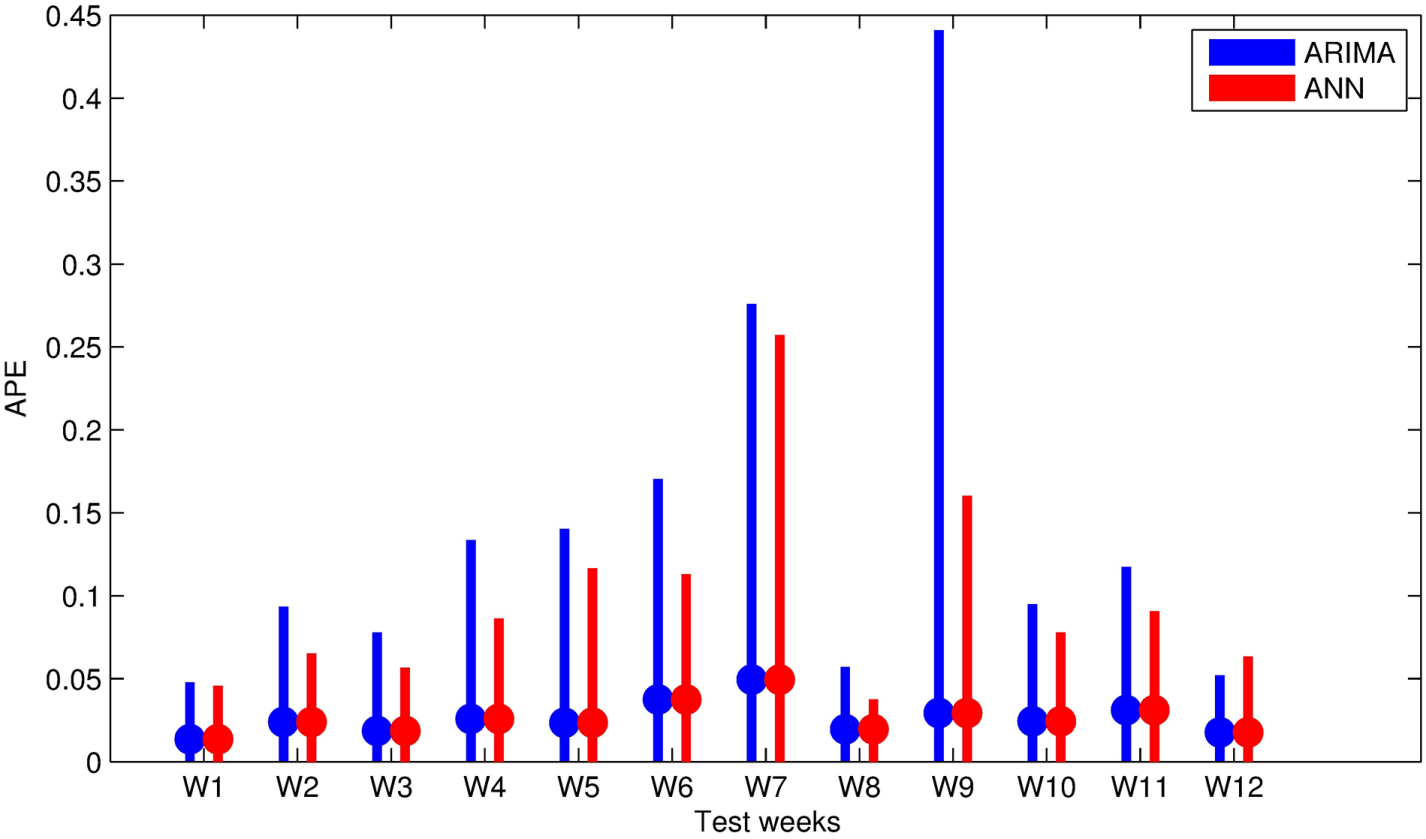
Performance comparison of the proposed approaches, measured with APE. MAPE values are highlighted on min-max intervals. Smaller values mean higher forecast accuracy.

**Fig 9:**
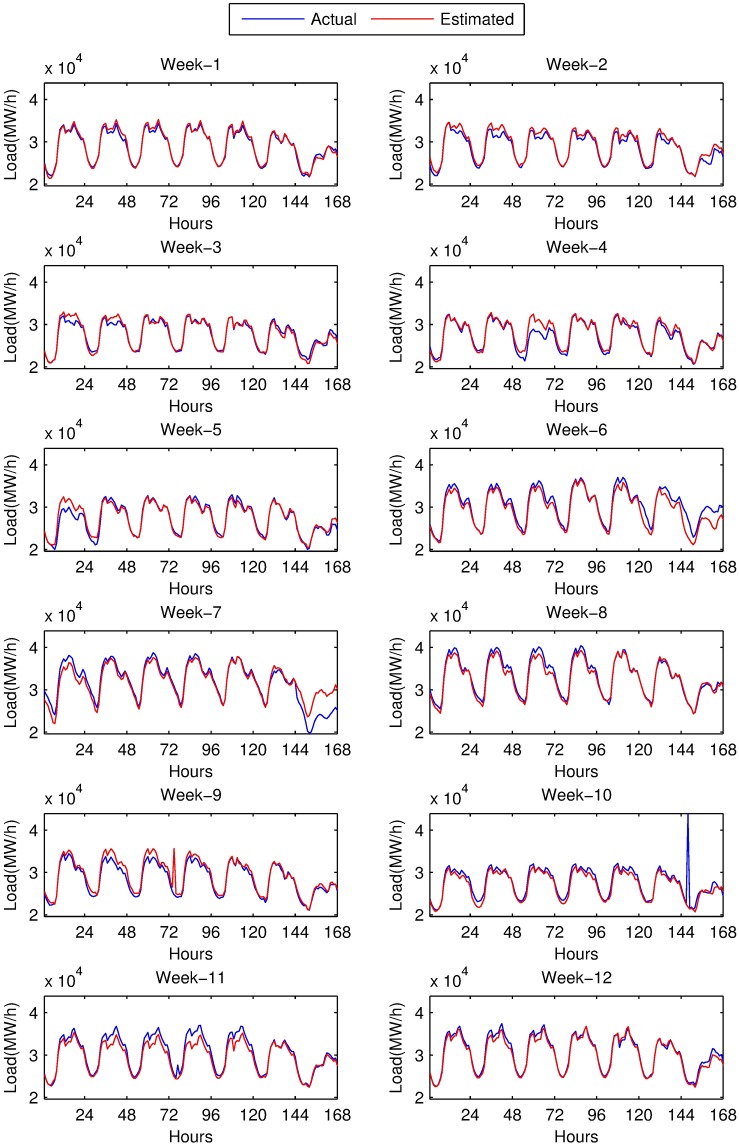
Load estimations of SARIMA based model and actual load values for 12 weeks of year 2014.

**Fig 10:**
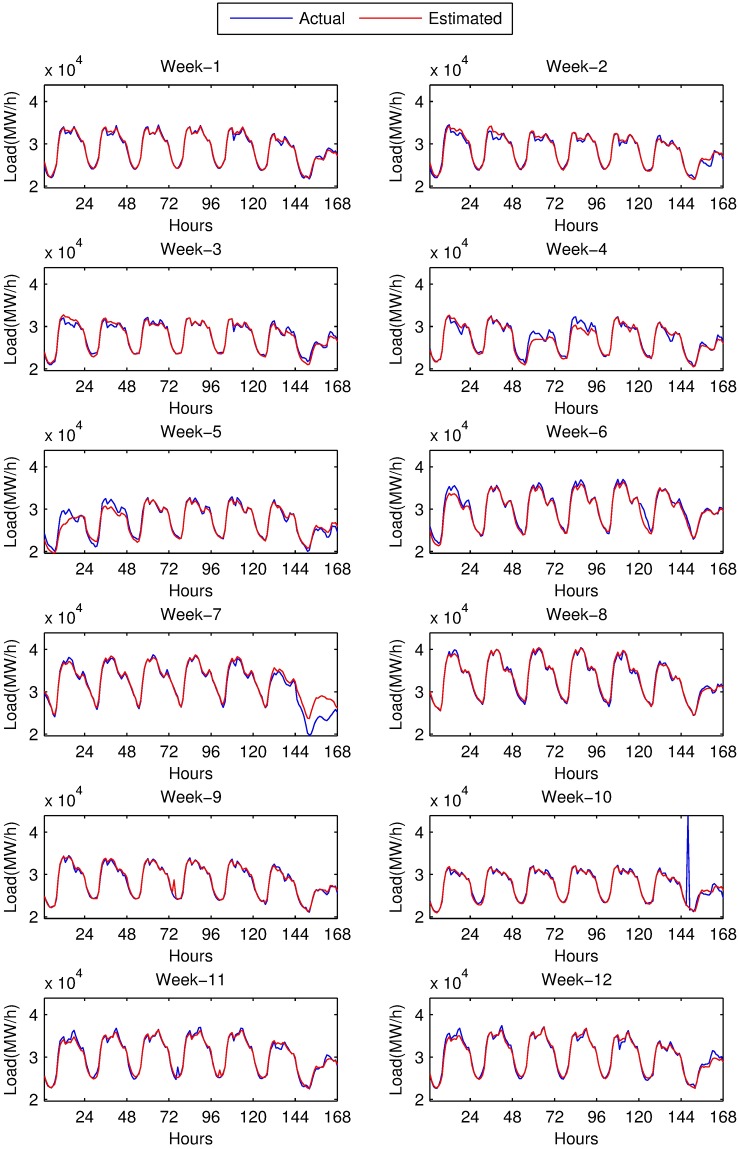
Load estimations of ANN based model and actual load values for 12 weeks of year 2014.

Performance evaluation of both methods is summarized in Table 8. The table is also visualized in Fig 8 using minimum, maximum APE values and MAPE values of each test week. Values in Table 8 and Fig 8 are calculated by averaging of 20 runs, in order to suppress the sensitivity of ANN to initial state. Hourly predictions of both models for 12 test weeks are also given in Fig 9 and in Fig 10. Our models predict all 168 hours of each test week at once. Therefore, we have a 168-hour ahead forecast horizon.

**Table 8:** Performance of implementations, measured with MAPE (%). Smaller values mean higher forecast accuracy.

Method	Evaluation	Week											
		1	2	3	4	5	6	7	8	9	10	11	12
SARIMA	Min APE	0.02	0.02	0.00	0.01	0.04	0.02	0.00	0.01	0.03	0.03	0.02	0.03
	Max APE	4.79	9.34	7.79	13.35	14.02	17.05	27.58	5.7	44.09	9.48	11.75	5.19
	MAPE	1.36	2.38	1.84	2.57	2.34	3.74	4.95	1.95	2.92	2.43	3.1	1.75
ANN	Min APE	0.00	0.02	0.01	0.01	0.01	0.00	0.01	0.01	0.00	0.00	0.02	0.00
	Max APE	4.58	6.53	5.66	8.62	11.65	11.3	25.73	3.76	16.03	7.78	9.07	6.33
	MAPE	0.98	1.42	1.45	2.01	2.26	1.81	3.26	1.03	1.03	1.13	1.38	1.36

Table 8 is obtained disregarding a special hour: MAPE of 10^th^ week is evaluated by removing the 146^th^ hour, which corresponds to October 28^th^, 2014 02:00 am. This hour was, unfortunately, end of daylight saving time, and since clocks are turned backwards from 2 am to 1 am, 2 am has occurred twice. Therefore, load of this hour is doubled. Clock change happens twice a year; start of daylight saving time, there happens a single hour with 0 load, and end of daylight saving time, where load is almost doubled for a single hour. We chose to disregard those hours.

At the first glance, it can easily be seen that performance of both methods depends on the season: average forecasting error for winter weeks is much less then that of summer, while spring and autumn are placed in between. Special cases for unexpected errors are explained below.

The highest forecasting error is at 9^th^ week which covers 22^nd^ to 28^th^ of September. Here, for a single hour, 75^th^ hour of the week (September 25^th^, 2014 02:00 am) both methods make an unfortunate peak causing the maximum error of the week. The cause of this peak is a inexplicable peak at same hour of previous week of the input data. The magnitude of the peak with SARIMA is reasonably greater than that of the ANN. Here the effect of seasonality on the model is revealed. ANN can smooth the noisy values, however, noise is directly reflected to forecasts.

Worst forecasted week seems to be the 7^th^ week according to the MAPE values. Forecasting error for the last day of this week is above the expectations. This day is not only Sunday but also Ramadan Feast Eve. Load during this day is about 20% less than an ordinary Sunday. The error occurred for this day spoils the MAPE of the week.

The 4^th^ and 5^th^ weeks include national days. April 23^rd^ is National Sovereignty and Children’s Day, and May 19^th^ is Commemoration of Atatürk, Youth and Sports Day. Those days not only cause errors on forecasting but also cause a noise in the training data and increase the error on the forecasting of 6^th^ week.

At the worst case, for week 9, forecasting error with ANN is 16.03% while highest forecasting for SARIMA model is 44.09%. The reason of this error was explained above as the noise in input. When overall performance of the methods is considered, ANN with calendar data and previous load outperforms SARIMA although the error trend seems to be the same. SARIMA’s main weakness is that there is no way to distinguish between the working days and holidays. Separate models for working days and holidays might be regarded as a solution. However, there are two religious holidays and four national holidays, which occur once a year. Religious holidays shift 10 days each year. It is also possible for all holidays to be extended by the government, if the holiday is close to weekend. Therefore separate models for SARIMA is not applicable and ANN is the method which provides the distinction required by the nature of electric consumption.

SARIMA’s benefit seems to be quick recovery from the effect of the holidays. The effect of national days in third day of 4^th^ week and first day of the 5^th^ week is reflected to next day in ANN model, however, SARIMA does not propagate this unexpected effect to next day. For the fourth day of 4^th^ week, ANN has 3.4% MAPE while ARIMA has 1.6% MAPE. Difference for the second day of 5^th^ week is not this much notable; MAPE values are 4.1% for ANN and 3.1% for SARIMA.

**Fig 11:**
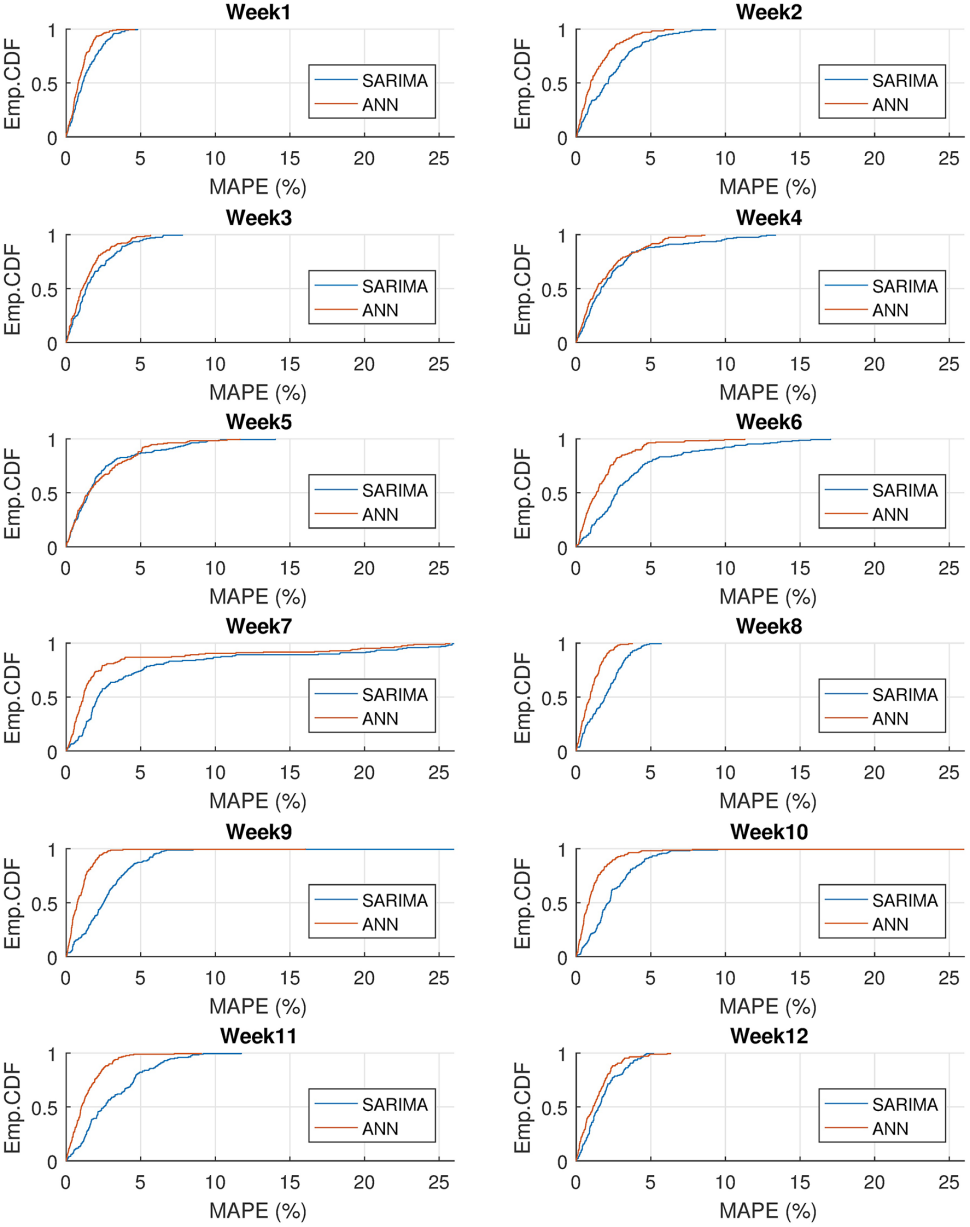
Empirical cumulative distribution functions for MAPEs of SARIMA and ANN based models on 12 test weeks of year 2014.

We compare the distribution of errors for the SARIMA and ANN based models in Fig 11 by using the empirical cumulative distribution function. We see that ANN based model produces less error than the SARIMA based model on most of the test weeks and the cumulative error is below 5% in general. However, SARIMA has minor advantages in some points of the 4^th^ and 5^th^ test weeks. This is due to the SARIMA’s ability to recover quickly from the effects of the national holidays in these months, as we discussed above.

## Conclusion

In this study, we created two separate STLF models based on SARIMA and ANN for Turkish Electricity Market. We comparatively presented their performances for last weeks of each month. On contrary to existing studies, we included weekends and special days in our test sets for fair and unbiased performance evaluation. Additionally, we evaluated the contribution of globally known factors on forecast performance, such as electricity price, weather parameters and currency.

When the model performances are observed on average of 12 test weeks, ANN produced 1.80% MAPE and outperformed SARIMA, which had 2.60% MAPE. We can say that ANN model fits better than SARIMA to Turkish Market. However, in some cases SARIMA performs better than ANN, especially on the forecasts after holidays. This structure addresses one of our future works, to produce a hybrid load forecast solution.

Experimental results proved that when more features are utilized, model becomes more complex and forecast performance decreases. For this reason, we do not recommend using load, price, weather and currency feature sets together. Calendar data and load feature sets work best on ANN for forecasting with adequate precision.

Our future work consists of building a hybrid model to produce more accurate forecasts, using the models and directive discussions we presented here. Moreover, we will use the output of this system as input to a short term electricity price forecaster. We also plan evaluating our model after the total liberalization of Turkish Market.
